# *Sauromatum guttatum* extract promotes wound healing and tissue regeneration in a burn mouse model *via* up-regulation of growth factors

**DOI:** 10.1080/13880209.2019.1676266

**Published:** 2019-10-25

**Authors:** Ali Said, Fazli Wahid, Kashif Bashir, Hafiz Majid Rasheed, Taous Khan, Zohaib Hussain, Sami Siraj

**Affiliations:** a Institute of Basic Medical Sciences, Khyber Medical University, Peshawar, Pakistan;; b Department of Biotechnology, COMSATS University Islamabad, Abbottabad, Pakistan;; c Department of Pharmacy, COMSATS University Islamabad, Abbottabad, Pakistan

**Keywords:** Re-epithelialization, burn wound healing, platelet-derived growth factor, epidermal growth factor, fibroblast growth factor

## Abstract

**Contexts:** *Sauromatum guttatum* (Wall.) Schott (Araceae) has been traditionally used for the treatment of wounds.

**Objectives:** This study evaluates the healing and tissue regeneration potential of *S. guttatum* extract in burn wounds.

**Materials and methods:** *S. guttatum* extract was analysed using various chemical tests, thin layer chromatography (TLC) and high-performance liquid chromatography (HPLC). Moreover, the extract was tested against burn associated bacteria and minimum inhibitory concentration (MIC) was also calculated. Wound healing and tissue regeneration potential was assessed using a thermally induced burn BALBc mouse model. *S. guttatum* extract (2% *w*/*w*) prepared in petroleum jelly, vehicle and positive control [silver sulfadiazine (SD)] groups was applied three times a day. The treatment was continued for 15 d and wound closure was measured and photographed on day 5, 10 and 15. The burnt tissues excised from wounds were subjected to histological and comparative gene expression analysis.

**Results:** The results of the chemical tests indicated the presence of alkaloids, saponins, phenols, phytosterols, tannins, and flavonoids, while TLC and HPLC analysis indicated the presence of various compounds. The extract showed excellent activity against the tested pathogens. The lowest MIC (125 µg/mL) was observed against *Staphylococcus aureus*. A considerable decrease in wound area (72%) was observed in extract-treated group. Histological examination of extract-treated group showed good signs of wound healing with complete re-epithelialization and better tissue regeneration. Comparative gene expression analysis revealed the up-regulation of wound healing related *PDGF*, *EGF* and *FGF* genes.

**Conclusions:** *S. guttatum* extract may be used to isolate bioactive constituents for the treatment of burn wounds.

## Introduction

Burns are the most common type of traumatic injuries (Ye and De 2017), causing morbidity, mortality or long-term disabilities in millions of people across the world (Priya et al. 2002). It has been estimated that approximately 265,000 deaths occur every year around the globe due to burn-related injuries (Karunanidhi et al. 2017). Approximately, 90% incidences are reported in low-income countries and almost 50% of them were recorded in South Asia (Dissanaike and Rahimi 2009). Despite modern health care services and novel dressing systems, burn-related death rates are increasing every day (Karunanidhi et al. 2017). Burn wound patients are prone to the cutaneous and systemic bacterial infections (Church et al. 2006) that can seriously delay the wound healing process and lead to scar formation (Annan and Houghton 2008). The site of burn wound contains dead tissue and protein-rich exudate that provides a suitable environment for the proliferation and colonization of microbes (Church et al. 2006). The burn wounds can be colonized by pathogenic strains of bacteria such as *Staphylococcus aureus*, *Salmonella typhyi, Pseudomonas aeruginosa, Bacillus cereus, Klebsiella, Escherichia coli,* methicillin-resistant *S. aureus* (MRSA) and many others (Church et al. 2006; Taneja et al. 2013). Silver sulfadiazine (SD) is one of the most widely used drugs for the treatment of second- and third-degree burns. SD possesses broad-spectrum activity against various burn associated pathogens but disadvantages like poor penetration to eschar and the ability to cause leukopoenia can limit its use (Karunanidhi et al. 2017). Moreover, the emergence of antibiotic resistance phenomena have inspired researchers to find alternative treatment options for burn associated and other forms infections.

Burn wound healing is a multi-factorial biological process which involves a complex series of cellular and molecular events (Yao et al. 2017). It is characterized by hemostasis, inflammation, cell division, re-epithelialization, collagen deposition and remodelling of the extracellular matrix (Adetutu et al. 2011). These events are regulated by various cellular and chemical mediators such as cytokines, inflammatory cells, growth factors and their inhibitors (Yao et al. 2017). Growth factors such as platelet-derived growth factor (PDGF), epidermal growth factor (EGF), fibroblast growth factor (FGF), insulin-like growth factor (IGF), vascular endothelial growth factor (VEGF) and transforming growth factor (TGF) play a crucial role in wound healing and tissue regeneration (Bae et al. 2014; Freiesleben et al. 2017). These factors attract skin cells towards the wound site, initiate cell proliferation and ultimately help in tissue repair (Bao et al. 2009). It has been reported that aberrant production of growth factors can seriously delay the wound healing process and may produce scars (Zarei and Soleimaninejad 2018). Since ancient times, physicians have been looking for alternatives that may have superior wound healing properties with inherent antimicrobial potential, less side effects and greater cost-effectiveness. In particular, natural products are more attractive as these can accelerate wound healing by stimulating release of various growth factors and cytokines. The natural products may have less side effects represent a costeffective process (Karunanidhi et al. 2017).

Since pre-historic time, various parts of medicinal plants have been used to combat skin related problems (Jridi et al. 2017). Furthermore, several plant extracts are well known in dermo-pharmacy that promote wound healing process through multiple molecular mechanisms (Bouassida et al. 2017). *Sauromatum guttatum* (Wall.) Schott (Araceae) is a garden plant commonly known as ‘Voodoo lilly or Snake Plant’ (Shah et al. 2014). The Araceae family has 105 genera and over 3300 species, some of them are grown as crops and for interior decorative purposes. Most of these species are indigenous to Pakistan, Malaysia, Indonesia, tropical Africa and America (Frausin et al. 2015). It has been reported that *S*. *guttatum* has alkaloids and tannins (Shah et al. 2014). Similarly, various phytochemicals have been isolated, including lectins, dimethyl sulphide, dimethyl tetrasulphide, β-caryophyllene, indole, skatole, ammonia, trimethylamine and primary amines (Irshad et al. 2012).

*S. guttatum* is traditionally used in the treatment of various health-related problems, such as asthma, diarrhoea, colic, flatulence, spasm and many others (Singh Bains et al. 2005; Shah et al. 2014). Traditionally, different plant species of Araceae family have been used for the treatment of insect bites, tumour and wound healing in many Asian countries. Moreover, some pharmacological activities of the Araceae family reported include antimicrobial, antimalarial, antispasmodic, anticancer, vasomodulator and bronchodilator (Frausin et al. 2015).

However, a literature review showed that no pharmacological reports are available on the burn wound healing and tissue regeneration effects of *S. guttatum.* Therefore, this study was carried out to assess the burn wound healing and tissue regeneration potential of *S. guttatum*. The results showed that *S. guttatum* extract has the ability to control burn associated infection, promote wound healing through upregulation of growth factors and enhance tissue regeneration.

## Materials and methods

### Plant materials

The tubers of *S. guttatum* were collected from Nathia Gali Abbottabad, Khyber Pakhtunkha, Pakistan during the month of July in 2016. The plant material was identified and authenticated by the plant taxonomist Dr. Abdul Nazir, Department of Environmental Sciences, COMSATS University Islamabad, Abbottabad Campus, Pakistan. A voucher specimen (CUHA-112) of the plant material was deposited at the herbarium unit in the respective department.

### Chemicals

Bacteriological nutrient agar and broth were purchased from Merck Ltd. (Darmstadt, Germany). Dimethyl sulphoxide (DMSO), *n*-hexane, methanol, formaldehyde, sodium phosphate monobasic (NaH_2_PO_4_), haematoxylin, paraffin wax was bought from Sigma-Aldrich (St. Louis, MO). Petroleum jelly, double distilled water, xylazine HCl, lignocaine HCl and ketamine HCl were procured from a local medical store.

### Processing and extraction of plant material

The collected plant material was washed, sliced into small pieces and shade dried at ambient temperature. The dried plant material was pulverized into a fine powder and macerated in 70% methanol at room temperature for 14 d with periodic stirring. The mixture was filtered through a muslin cloth and then passed through Whatman filter paper No. 42 (125 mm) to remove any solid residue. The process was repeated two times to fully exhaust the plant material. Then, vacuum rotary evaporator (Buchi Switzerland Rota vapour R-300 system) was used to concentrate the extract at 40 °C under reduced pressure. The extract was stored at 4 °C untill the use for further experimentation (Saeed et al. 2017).

### Phytochemical analysis and fingerprinting

Standard reported chemical tests were used to investigate the presence of major phytochemicals like flavonoids, saponins, phytosterols, phenols, tannins and alkaloids (Asad et al. 2007).

TLC analysis was performed to further confirm the presence of various major phytochemicals in the crude extract. TLC plates (Merck) pre-coated with silica gel (60 F254) were used as stationary phase, while methanol-chloroform (8:2 *v*/*v*) was used as mobile phase. TLC plate was developed in the above mentioned mobile phase and visualized under UV-light (254 or 366 nm) and ceric sulphate was sprayed on TLC plate to visualize spots of various constituents. The affinities of various constituents were measured in the form of retardation factor (R*f*) values using the formula below.

$$\text{R}f=\frac{\text{Distance}\;\text{moved}\;\text{by}\;\text{individual}\;\text{compound}\;}{\text{Total}\;\text{distance}\;\text{moved}\;\text{by}\;\text{mobile}\;\text{phase}\;}$$

High performance liquid chromatographic (HPLC) analysis was carried out using SHIMADZU HPLC system (LC-20AP pump) coupled with Prominence UV–VIS detector (SPD-20A/20AV). The Shim-Pack GIST C18 column (150 mm × 4.6 mm i.d.×5 μm) from SHIMADZU (Kyoto, Japan) was employed at 30 °C. Separations were made in isocratic mode using methanol:water (1:9; *v*/*v*) at a flow rate of 1 mL/min with an injection volume (loop) of 20 μL; UV detection was at 205 nm. The crude extract (5 mg/20 mL) was dissolved in HPLC grade methanol and filtered through 0.45 µm syringe before injecting into the HPLC.

### Antibacterial assay

*S. guttatum* extract was screened against various strains of Gram-negative and Gram-positive bacteria including *E. coli, P. aeruginosa, S. typhi, Klebsiella*, *S. aureus, B. cereus* and MRSA using agar well diffusion method (Khan et al. 2009). The overnight bacterial culture (200 μL) was uniformly spread on the surface of Mueller Hinton agar. After this, the required number of wells was made through pre-sterilized 1000 μL pipette tips. SD and DMSO were used as positive and vehicle controls, respectively. Then, 20 mg plant extract, dissolved in 1 mL of DMSO was loaded on agar well plates. Similarly, standard and DMSO were also applied and incubated at 37 °C for 24 h. Thereafter, the zone of inhibition was measured using standard procedure (Khalid et al. 2017).

### Minimum inhibitory concentration (MIC)

The MIC of the extract was assessed by using 96 well plates. The 12 wells of each row were filled with 95 μL of nutrient broth (Bussmann et al. 2010; Osaili et al. 2019). The row A was used as positive control containing media and inoculum. In row B, 5 μL plant extract and additional 95 μL media were added. Subsequently, the 100 μL of mixture from the row B was transferred to next well of row C for serial dilution. This serial dilution was continued to row G to create a concentration sequence from 1000 to 31.25 µg/mL. From row G, 100 μL was discarded to maintain the final concentration. Row H was taken as negative control containing media. In the end, 5 μL of bacterial culture were added into all wells except the negative control. The plates were incubated at 37 °C for 24 h. The activity was performed in triplicates for each bacterial species and MIC was calculated using the following equation.

$$\text{MIC}=\frac{\text{Lowest}\;\text{concentration}\;\text{of}\;\text{the}\;\text{extract}\;\text{inhibited}\;\text{the}\;\text{growth}+\text{highest}\;\text{concentration}\;\text{allowed}\;\text{the}\;\text{growth}}{2}$$

### Animals and induction of burn wounds

BALB^c^ mice are often used as an animal model for the wound healing activity (Dai et al. 2011). Therefore, BALB^c^ mice were used in this study. The animals were purchased from National Institute of Health (NIH), Islamabad, Pakistan and kept as per Khyber Medical University (KMU), Peshawar, Pakistan, research guidelines, which are in compliance with the NIH recommendations (1989). The animals were caged under standard conditions and free access to water and balanced diet. All procedures in this study were reviewed and approved by the KMU Ethical Committee for animal experimentations (No. DIR/KMU-EB/PA/000433).

A total of 20 animals were used and divided into four groups (five animals/group) according to the average weight (30.2 g ± 1.50). Burn wound was induced according to the previously described protocols (Agnihotri et al. 2004; Khalid, Khan, et al. 2017). Briefly, mice were anaesthetized through intra peritoneal injection of ketamine (100 mg/kg) and xylazine (10 mg/kg). Then, the dorsal area of the animals was shaved by using razor and hair removing cream. Alcohol swabs were used to disinfect the shaved area before inducing thermal injury. Partial thickness burn was induced by using specially designed metal bar, which was pre heated on opened flame. The metal bar was applied perpendicularly on dorsal area for 9 s to produce second-degree burn. The inflicted burn wound area was 225 mm^2^.

### Preparation and application of *S. guttatum* ointment and wound assessment

The topical ointment of *S. guttatum* extract (2% *w*/*w*) was prepared in petroleum jelly through geometric mixing. One group of animals was treated with the ointment of *S. guttatum* extract, while the other group was taken as vehicle control and treated with petroleum jelly. The positive control group received topical SD (1%) cream. One group was left untreated and considered as negative control. After wound induction plant extract ointment, vehicle and SD cream were applied topically three times a day for 15 d. Wound area was measured with a standard ruler and photographs were also taken on day 0, 5, 10 and 15 of the study.

### Histological examinations of the skin tissue

On the last day of the experiment, all animals were sacrificed through cervical dislocation and tissue specimens were excised from the wound site. The excised tissue samples were equally divided, half of which were used for histological analysis and the other half for polymerase chain reaction (PCR) analysis. For histological analysis, the tissues were preserved in 10% neutral buffered formalin at 4 °C. Then, samples were passed through a series of increasing concentration of alcohol (70%, 80%, 90% and 100%) for 2, 3 and 6 h, respectively. Pure xylene solution was used to remove alcohol and finally tissue sections were embedded in paraffin wax. Thin sections of tissue samples were cut with a microtome (Robus technology model RM 250, 2010) and stained with haematoxylin and eosin dye (H&E). The prepared slides were examined under a compound microscope equipped with a digital camera to check the progression and quality of wound healing in all groups (Khalid, Khan, et al. 2017).

### Comparative gene expression analysis

Reverse transcription-polymerase chain reaction (RT-PCR) was used to assess the expression of genes involved in wound healing. For this purpose, total RNA was isolated from frozen excised wound tissues using TRIzol as described by the manufacturer (Invitrogen, Carlsbad, CA). *PDGF*, *EGF*, *FGF* and internal reference glyceraldehyde-3-phosphate dehydrogenase (GAPDH) gene specific primers were designed as given in Table 1. RNA was quantified using standard A260/280 absorbance protocol with Nanodrop (Thermofisher, Waltham, MA). Total RNA (650 ng) was reverse transcribed according to the manufacturer instructions using cDNA synthesis kit (Thermo Scientific, Waltham, MA). The synthesized cDNA was stored at −20 °C for further PCR analysis. The PCR amplification conditions were set using the programme: initial denaturation at 95 °C for 5 min, followed by 35 cycles of 95 °C for 40 s, annealing at 58 °C for 30 s, elongation at 72 °C for 35 s and final elongation at 72 °C for 5 min. Then, PCR amplified products were resolved on 1.5% agarose gel having stained with ethidium bromide and images were captured using UV trans-illuminator.

**Table 1.** Nucleotides sequence of primers and amplicon size.

Genes	Forward primer sequence	Reverse primer sequence	Amplicon size (bp)
*GAPDH*	GAACGGGAAGCTCACTGGC	GCATGTCAGATCCACAACGG	70
*PDGF*	CTGGCTCGAAGTCAGATCCACA	GACTTGTCTCCAAGGCATCCTC	98
*EGF*	GAGTTCCGTACTCCCTCTTGCA	CAGCCAAGACTGTAGTGTGGTC	118
*FGF*	AAGCGGCTCTACTGCAAGAACG	CCTTGATAGACACAACTCCTCTC	139

### Statistical analysis

All quantitative data were expressed as the average means ± standard derivation while statistical analysis was performed by using Student *t-*test through sigma plot version 2001. *p* values ≤0.05 were deemed significant.

## Results

### Phytochemical analysis of *S. guttatum*

**Figure 1.**
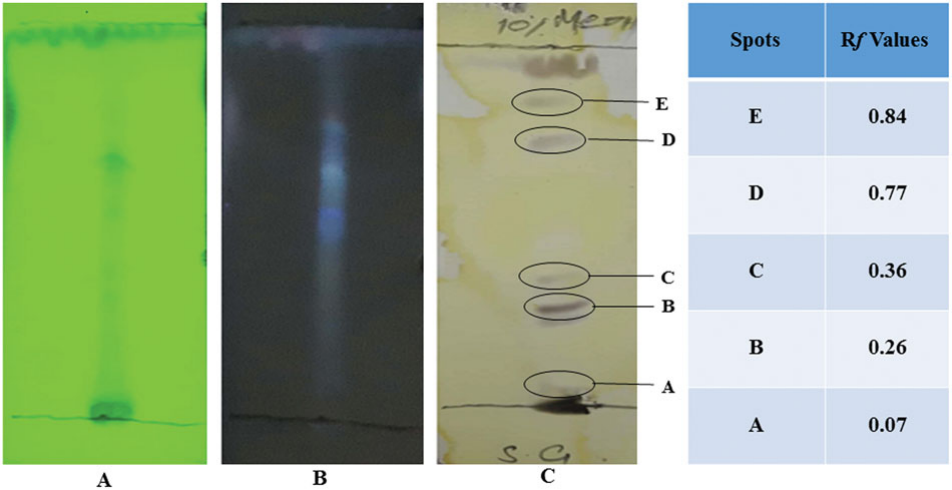
TLC profile of *S. guttatum* extract using silica gel coated TLC plates. Methanol and chloroform (8:2) were used as a mobile phase. (A) Image taken under short UV light 254 nm, (B) long UV light 366 nm and (C) image taken after spraying with ceric sulphate.

**Figure 2.**
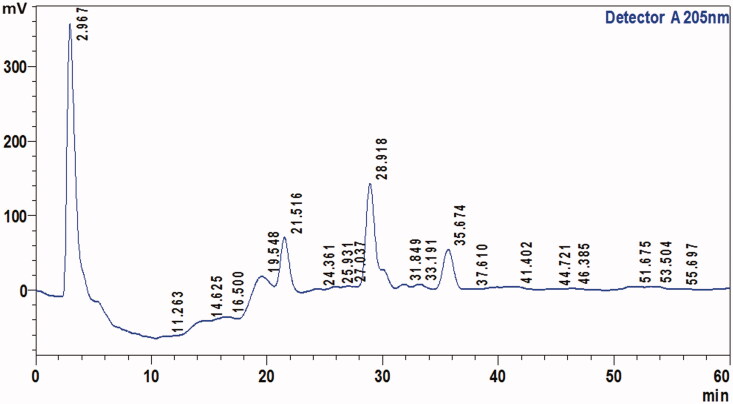
The HPLC chromatogram of methanolic extract of *S. guttatum* analysed under the HPLC conditions: mobile phase, methanol: water (1:9, *v*/*v*), wavelength (205 nm), flow rate (1 mL/min), injection volume (20 μL), Column: Shim-pack, octadecylsilane ODS (C_18_) analytical column (150 mm × 4.6 mm, 5 µm).

It is important to know the phytochemical composition of any plant extract before evaluating its pharmacological activities. Therefore, the chemical composition of *S. guttatum* crude extract was evaluated. The results indicated the presence of flavonoids, saponins, phytosterols, phenols, tannins and alkaloids, but glycosides and proteins were not detected as shown in Table 2. To confirm the presence of various compounds in the extract, TLC analysis was carried out. The TLC chromatogram and retention factor (R*f*) values of various compounds from A to E of the extract are shown in Figure 1. TLC chromatogram of crude extract showed various spots and *Rf* values as shown in Figure 1 are A (0.07), B (0.26), C (0.36), D (0.77) and E (0.84). HPLC analysis of the crude extract derived from *S. guttatum* showed the appearance of four major and various minor peaks (Figure 2). The major peaks appeared at retention time of 2.96, 21.5, 28.9 and 35.67 min.

**Table 2.** Phytochemical analysis of *S. guttatum* extract.

Phytochemical constituents	Chemical test	Presence
Flavonoids	Lead acetate	+
Saponins	Foam	+
Phytosterol	Salkwoski’s	+
Phenol	Ferric chloride	+
Tannins	Gelatin	+
Alkaloids	Dragendorff’s	+
Glycosides	Modified Borntrager’s	−
Amino acids	Ninhydrin test	−

Definition: + indicates the presence; − indicates absence of phytochemicals.

### Antimicrobial assay of *S. guttatum*

The antimicrobial activity of *S. guttatum* extract was checked against various burn associated pathogens. Results showed that *S. guttatum* inhibited the growth of *E. coli, P. aeruginosa*, S*. typhi*, *Klebsiella*, *S. aureus*, *B. cereus* and MRSA with zone of inhibition of 11, 11, 16.3, 11, 20, 12.5 and 11 mm, respectively. The MIC values of the extract against *E. coli, P. aeruginosa*, S*. typhi*, *Klebsiella*, *S. aureus*, *B. cereus* and MRSA were 187.5, 250, 312.5, 250, 125, 187.5 and 375 µg/mL, respectively.

### Burn wound healing activity of *S. guttatum*

**Figure 3.**
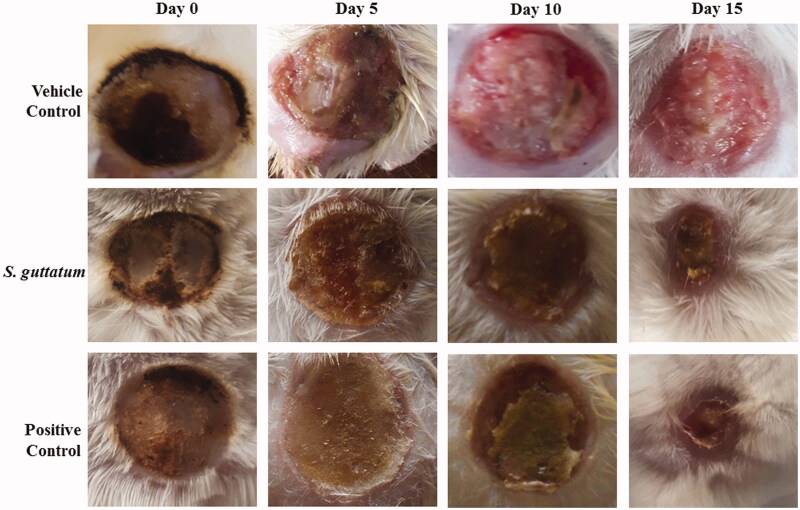
Representative photographs of wounds of vehicle treated, *S. guttatum* extract (2%) and silver sulfadiazine (positive control) treated groups on day 0, 5, 10 and 15.

In this study, the burn wound healing activity showed wound narrowing from 225 ± 0 mm^2^ (day 0) to 63 ± 8.28 mm^2^ (day 15) in *S. guttatum* treated group (Figure 3; Table 3). In comparison, the vehicle-treated group wound area was reduced from 225 ± 0 mm^2^ up to 197 ± 5.09 on day 15. The wound area of the positive control group reduced from 225 ± 0 mm^2^ (day 0) to 41 ± 1.41 mm^2^ (day 15), which was close to the *S. guttatum* treated group (Table 3).

**Table 3.** The average wound closure area on different days during the tretment of *S. guttatum* extract, vehicle and positive control groups.

Group name	Average burn wound area in mm^2^			
	Day 0	Day 5	Day 10	Day 15
Vehicle Control	225 ± 0	386.3 ± 18.62	290 ± 14.14	197 ± 5.09
*S. guttatum*	225 ± 0	256 ± 13.05	162.3 ± 12.28***	63.0 ± 8.28***
Positive Control	225 ± 0	251 ± 7.07	156.6 ± 0.94	41 ± 1.41

*p* Value ≤ 0.05 was considered to be statistically significant (*denotes significant statistical differences, ***p ≤ 0.001).

### Histological analysis of skin tissues

**Figure 4.**
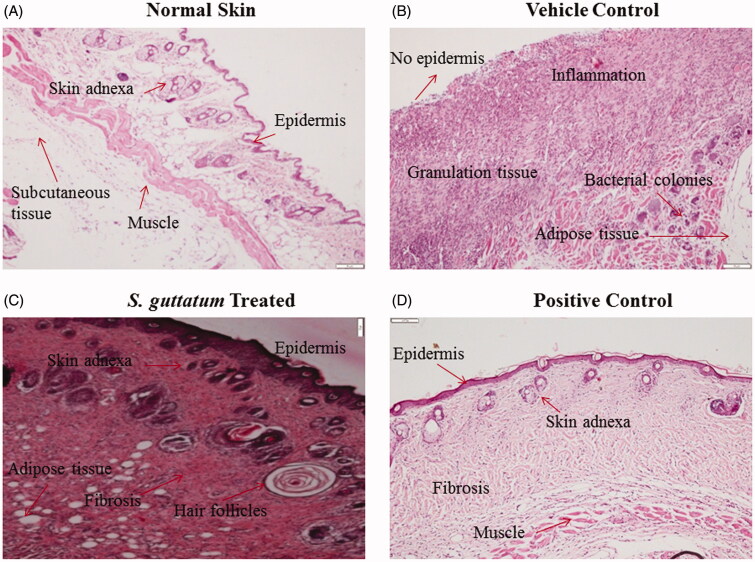
Histological images of (A) normal skin and burnt skin tissues treated with (B) vehicle, (C) *S. guttatum* extract and (D) positive control.

In histological examination, images of haematoxylin and eosin (H&E) stained tissues of each group are displayed in Figure 4. Normal epithelial regeneration with formation of blood vessels, granulated tissue and sebaceous glands can be seen in the images of *S. guttatum* extract treated tissues (Figure 4). In addition, the formation of hair follicles indicated that normal healing has occurred. The similar healing process was also observed in positive control group.

### Comparative gene expression analysis

**Figure 5.**
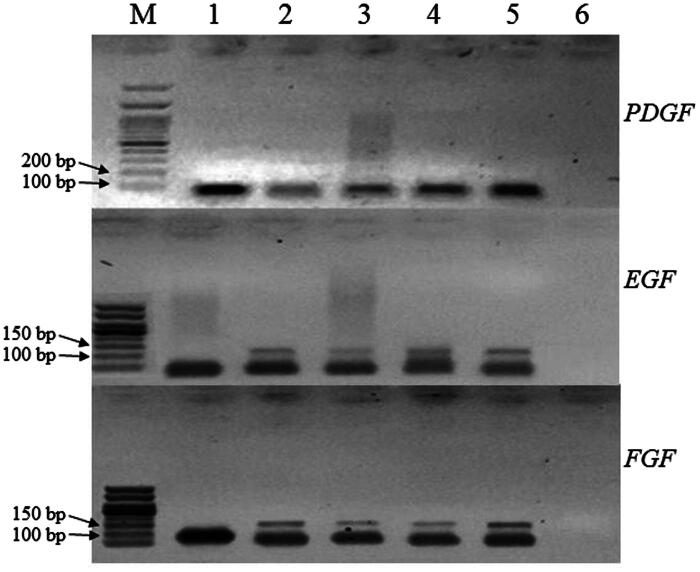
Comparative gene expression analysis of important growth factors involved in regulation of wound healing and regeneration. The skin tissue of the animals treated with *S. guttatum* showed increased expression of *FGF*, *EGF* and *PDGF* genes as compared to vehicle control. M lane: Marker, Lane 1: GAPDH, Lane 2: Positive Control (silver sulfadiazine), Lane 3: Vehicle control, Lane 4: Untreated, Lane 5: *S. guttatum* and Lane 6: Water.

During the process of cutaneous wound healing, various growth factors, cytokines and chemokines play a role in regulation and modulation of wound closure. The results showed that expression of PDGF, EGF and FGF increased in response to *S. guttatum* extract treatment as compared to vehicle and untreated group as shown in Figure 5. Similarly, the positive control group also showed upregulation of the selected genes.

## Discussion

*S. guttatum* is traditionally used in inflammation and wound healing. However, literature lacks the scientific investigation of *S. guttatum* in burn wound healing. For this, it is important to scientifically investigate the chemical composition of plant extract. Therefore, this study was carried out to scientifically assess the potential of *S. guttatum* extract as an alternative treatment in burn wound healing. Phytochemical analysis through chemical tests, TLC and HPLC was also performed in this study.

In chemical tests for phytochemical analysis, results indicated the presence of all major chemical constituents except proteins. There was limited literature available on the *S. guttatum* phytochemical composition; however, studies reported that *S. guttatum* contains alkaloids, tannins and lectins (Shah et al. 2014) that are in accordance with the current results. TLC analysis exhibited the presence of various chemical constituents. The literature lacks evidence for the TLC of *S. guttatum* extract; therefore, this information can be used to isolate new compounds from *S. guttatum* extract in future. In HPLC analysis, four major peaks were identified but the literature has limited evidence for the HPLC results, however, previously size exclusion and cation exchange HPLC was used to purify lectin from tubers of *S. guttatum* (Shah et al. 2014). It can be concluded from the phytochemical analysis that *S. guttatum* extract may have various bioactive compounds. In the future, the information from this data may be used to isolate and identify the healing related bioactive compounds.

Disruption of skin after burn injury and subsequent immune suppression favour microbes to colonize the wound and interfere with healing process. *S. aureus,* and *P. aeruginosa* are the highly prevalent strains (Agnihotri et al. 2004), while MRSA*, E. coli, B. cereus* and *Klebsiella* are also largely associated with burn wounds (Khalid, Ullah, et al. 2017; Sajjad et al. 2019). The presence of resistant strains may lead to chronic non-healing wound, which prolongs the hospital stay. Therefore, burn associated bacterial strains were used in this study and it is evident from the zone of inhibition results that *S. guttatum* extract was even more effective than standard drug against *S. typhyi* and *B. cereus* while having comparable activity with the standard drug against *Klebsiella*, *S. aureus* and MRSA. However, the MIC data show that extract was effective against all tested strains except *S. typhyi* and MRSA. It is suggested that presence of phenolic compounds in *S. guttatum* extract may be responsible for inhibiting the microbial growth. These results are in accordance with previous literature where flavonoids, saponins and tannins were reported as antimicrobial agents (Cowan 1999; Shimada 2006; Patel 2014). As *S. guttatum* is active against broad-spectrum of bacteria so it can be used to control burn associated infection that may ultimately augment the wound repair process.

Burn wound healing is a complex physiological process through which damaged skin tissue regains the normal anatomy after thermal injury (Rowan et al. 2015). During the healing process, keratinocytes and epidermal cells from wound edges proliferate inward and contract the wound size (Shukla et al. 1999). In burn wound analysis, results showed that *S. guttatum* extract promotes sustainable wound healing pattern. Plant extract can accelerate wound healing process through multiple mechanisms such as by upregulating various growth factors, cell division, maturation and migration of various cells involved in healing (Shukla et al. 1999). Unfortunately, limited data are available for the mechanism of wound healing by plants of the Araceae family. However, it may be suggested that keratinocytes, epidermal cells and fibroblasts were activated by the *S. guttatum* extract to accelerate the healing process.

Histological examinations of all groups were performed (Khalid, Ullah, et al. 2017) to check the tissue regeneration and wound healing progression. The results are in accordance with previous studies, which show more granulation tissues in the dermis along with fibroblast and new blood vessels at day 15 of normal healing process (El-Sayed 2016). It is also apparent from the literature that plant extract and isolated compounds may support the repair process of the skin (Karunanidhi et al. 2017). It is important to mention that incomplete epithelial regeneration, ulceration slough and necrotic tissues can be seen in vehicle-treated group. In high power images, numerous bacterial colonies, inflammatory cells and immature fibroblasts were also observed in vehicle-treated group. All these signs show that poor healing occurred in vehicle-treated group. Based on the histological observations, it can be concluded that *S. guttatum* extract promotes proper regeneration of the burnt tissues.

PDGF, EGF and FGF are the predominant growth regulators in healing (Bae et al. 2014). During the wound healing process, PDGF is involved in chemotaxis of inflammatory cells to clear the dead cells and pathogens from wound site (Seppä et al. 1982; Barrientos et al. 2008). Next, in proliferation EGF initiate signals to migrate and proliferate new skin cells (fibroblast, keratinocyte and epidermal cells) (Barrientos et al. 2008). At the end, FGF modulates fibrosis and remodelling along with improving the tensile strength of new tissues (Diegelmann and Evans 2004; Freiesleben et al. 2017). As shown in Figure 5, in comparison to vehicle, all the three genes were up-regulated in extract treated group. This means a heightened regenerative and healing potential in the treated group, owing to multiple mechanisms and pathways as has been described above. Therefore, it is suggested that *S. guttatum* extract may enhance wound healing process at different stages through up-regulation of *PDGF*, *EGF* and *FGF* genes.

## Conclusions

Based on the results, it can be concluded that *S. guttatum* extract has various classes of phytochemicals, especially flavonoids and alkaloids that may have a role in microbial control and wound healing. In the future, bioactive compounds can be isolated from *S. guttatum* that may be active against burn associated pathogens and enhance wound healing and tissue regeneration. These findings also suggest that *S. guttatum* may serve as an important component in wound treatment materials such as wound dressing or gels.
